# Microbiological colonization of peripheral venous catheters: a prospective observational study in a Swedish county hospital

**DOI:** 10.1016/j.infpip.2021.100152

**Published:** 2021-06-07

**Authors:** D. Juhlin, F. Hammarskjöld, S. Mernelius, K. Taxbro, S. Berg

**Affiliations:** a Department of Anaesthesia and Intensive Care Medicine, Ryhov County Hospital, Jönköping, Sweden; b Department of Cardiothoracic Anaesthesia and Intensive Care Medicine, Linköping University Hospital, Linköping, Sweden; c Microbiology Laboratory, Department of Laboratory Services, Division of Medical Services, Ryhov County Hospital, Jönköping, Sweden; d Faculty of Medicine and Health Sciences, Linköping University, Linköping, Sweden

**Keywords:** Peripheral venous catheter, Microbiological colonization, Injection port, Infection control

## Abstract

**Background:**

Most peripheral venous catheters (PVCs) used in Scandinavia are fitted with an injection port, creating an open PVC system. This port is difficult to disinfect, which may lead to the introduction of micro-organisms upon use.

**Aim:**

To investigate the prevalence of microbiological colonization of the injection port and internal lumen of ported PVCs with a minimum dwell time of 48 h at sample collection.

**Methods:**

Adult patients admitted to different medical and surgical departments and the intensive care unit were invited to participate in this prospective observational study. With the PVC *in situ*, the injection port and internal lumen were swabbed and cultured separately. Demographic and clinical data were collected to compare patients with colonized and non-colonized PVCs.

**Findings:**

In total, 300 PVCs from 300 patients were analysed. Of these, 33 patients (11.0%) had at least one positive culture. The colonization locations were as follows: port only, 26 (8.7%); internal lumen only, 5 (1.7%); and port and internal lumen, 2 (0.7%). The colonization rate was significantly higher in the injection port than in the internal lumen (*P*<0.0001). A ported PVC inserted in the hand incurred a significant risk of colonization (*P*=0.03). The odds ratio for colonization among patients in the infectious diseases department was 0.1 (95% confidence interval 0.1–1; *P*<0.06) compared with patients in the medical department.

**Conclusion:**

This study showed that 11% of ported PVCs were colonized by micro-organisms, with the vast majority (8.7%) of colonization occurring in the injection port.

**Clinical trial registration:**

ClinicalTrials.gov; ID NCT03351725.

## Introduction

Peripheral venous catheters (PVCs) represent 80–95% of all intravascular catheters used in humans [1]. Some of the most problematic complications due to PVCs are phlebitis, thrombosis and infection [2]. PVC-related (PVCR) infections cause morbidity, mortality and increased healthcare costs [3,4]. In clinical practice, a PVCR bloodstream infection (BSI) may be overlooked when thrombophlebitis or cultures from PVCs are absent. The incidence of PVCR-BSI has been reported to be 0.1% or 0.5 per 1000 catheter-days [5].

The absolute risk for PVCR-BSI is probably lower than that for central venous catheters; however, because of the widespread use of PVCs, many patients are exposed to the risk of harm [5]. Multi-modal preventive strategies have shown a sustained reduction in PVCR-BSIs with decreased morbidity and mortality [4,6]. Measures typically applied for prevention include continuous surveillance of PVCR-BSIs, training of healthcare workers, use of sterile gloves, upgradation of skin antisepsis, and introduction of closed intravenous catheter systems.

**Figure 1:**
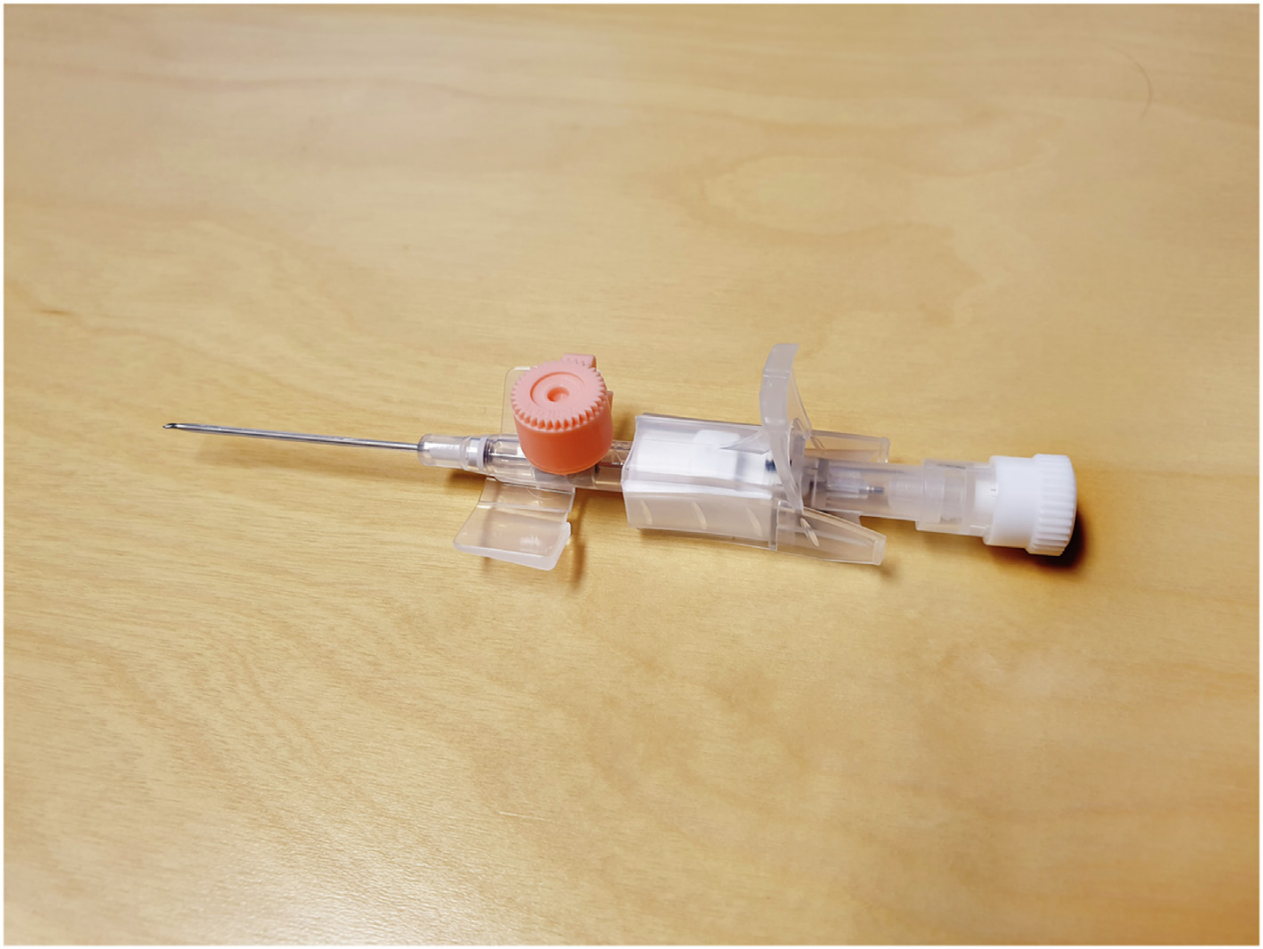
A ported peripheral venous catheter used in Scandinavia. The injection port is located under the pink cap and the internal lumen is located inside the white cap.

Regarding the introduction of closed intravenous catheter systems, there is an ongoing debate on how a PVC is best constructed to minimize the risk of PVCR-BSIs [7]. Most PVCs used in Scandinavia are fitted with an injection port (Figure 1) rather than a closed intravenous catheter system. The injection port is difficult to disinfect because of its design, which comprises an elevated and narrow plastic rim surrounding an injection membrane. This may lead to the injection of micro-organisms when a PVC is used. However, needleless connectors (NCs) that function as an alternative when using closed PVCs are prone to microbiological colonization, and need to be disinfected meticulously prior to use [8]. Several studies have shown problems with adherence to this routine [9].

The aim of this in-vivo study was to investigate the prevalence of microbiological colonization of the port and the internal lumen of ported PVCs with a minimum dwell time of 48 h at sample collection.

## Methods

### Setting

This study was conducted in a general public county hospital with 500 beds supporting most medical, oncological and surgical specialties, except cardiothoracic and neurosurgery.

### Study population

Patients aged ≥18 years who provided informed consent and were admitted to the medical, surgical or infectious disease departments or the intensive care unit (ICU) and had a PVC dwell time ≥48 h at sample collection were eligible for inclusion. A patient could only be included once in the study. If multiple PVCs were sampled from one patient at different times, only the first PVC sample was included. Exclusion criteria were as follows: the PVC was *in situ* for <48 h; the inner dimension of the PVC was <0.9 mm, 22 G; or an incomplete culture was obtained from a sample.

### Catheter design and insertion procedures

All PVCs in this study were polyurethane catheters from two different manufacturers (Becton Dickinson, Franklin Lakes, NJ, USA; B. Braun Medical AB, Melsungen, Germany). During the study period, the PVC insertion protocol included adequate implementation of basic hygiene routines, disinfection of the skin with 0.5% chlorhexidine gluconate in 70% isopropyl alcohol, use of high-purity gloves, and fixation with a transparent dressing. The protocol did not prescribe port disinfection prior to use. After insertion, the PVC type, site and time of insertion were registered in the patient's electronic medical record. Furthermore, PVC inspection was performed daily, and routine replacement was performed every 72 h, except in specific cases wherein the replacement was performed later than 72 h for clinical reasons.

### Data collection and microbiological methods

One nurse at each participating ward was trained to perform the procedures according to the study protocol. While the patient still had the PVC *in situ*, the injection port and internal lumen were swabbed with two separate sterile cotton-tipped swabs moistened with sterile sodium chloride (0.9%). The swabs were placed immediately in a collection tube containing Amies medium with charcoal, and transported to a local microbiological laboratory. The samples were cultured on haematin agar plates and incubated overnight at 37°C in air with the addition of 5% CO_2_. Species identification was performed using matrix-assisted laser desorption/ionization-time of flight mass spectrometry (Bruker, Billerica, MA, USA), according to the manufacturer's instructions. Cultures were categorized as ‘positive’ if at least one colony-forming unit of any bacteria was found.

If the same species of micro-organism was found in the port and the internal lumen, whole-genome sequencing was performed. DNA was extracted from isolated *Enterococcus faecium* and *Staphylococcus aureus* using the EZ1 DNA tissue kit on the EZ1 Advanced XL (Qiagen, Hilden, Germany). Library preparation was performed using Nextera XT library prep kit (Illumina, San Diego, CA) according to the manufacturer's instructions. Paired-end sequencing (2×250 cycles) was performed using a Miseq instrument (Illumina). Core genome multi-locus sequence typing (cgMLST) assembly and cluster analysis was performed using SeqSphere (Ridom GmbH, Münster, Germany). The cgMLST schemes were based on 1423 genes for *E. faecium* and 1861 genes for *S. aureus.*

All other data were collected manually for 2019 and 2020 from the patient's electronic medical record (Table I, Table Ⅱ, Table Ⅲ). The following data were analysed: age, sex, type of department (medical, surgical, infectious diseases, ICU), Charlson Comorbidity Index (CCI) [10], acute or planned admission to hospital, length of stay, insertion site, PVC size, dwell time at sample collection, time from admission to PVC insertion, if patient had a positive blood culture within ±72 h of PVC sample collection, if patient was given antibiotics due to a PVCR infection, if patient died from a PVCR infection, and if patient was immunocompromised according to the Acute Physiologic Assessment and Chronic Health Evaluation II (APACHE II) score [11].

### Ethics

This study was approved by the Regional Ethical Review Board of Linköping (2015/477-31).

### Registration

The study was registered on ClinicalTrials.gov; (ID NCT03351725; Release Date: 15^th^ November 2017).

### Statistical analysis

This was an exploratory investigation and colonization rates were not known *a priori*; as such, a sample size calculation could not be performed. Descriptive analyses were performed to characterize the patient population. Pearson's Chi-squared test, Fisher's exact test, Mann–Whitney *U*-test and Student's *t*-test were used to test for comparisons between groups, depending on whether the data were discrete or continuous, and whether distributions were normal. Logistic regression models were used to predict the odds of PVC colonization based on several potential risk factors. All *P*-values were two-tailed, and *P*<0.05 was considered to indicate significance. Data were analysed using SPSS Version 26 (IBM Corp., Armonk, NY, USA).

## Results

In total, samples were collected from 337 PVCs in 304 patients between May 2016 and January 2018. One patient was excluded due to protocol violation (age <18 years), one was excluded because the PVC had been *in situ* for <48 h, two were excluded because only incomplete cultures were obtained from their samples, and 33 PVCs were excluded because they came from patients who had already been included. Hence, 300 PVCs from 300 patients were analysed. Of these, 33 patients (11.0%) had at least one positive culture.

Patient characteristics are presented in Table I. The median dwell time at sample collection was 3 days (range 2–8 days). Time from hospital admission to PVC insertion was compared between the colonized and non-colonized groups, and no significant difference was found (*P*=0.22). Comparisons between colonized and non-colonized groups regarding various demographic and clinical factors are shown in Table II.

**Table I:** Patients' characteristics.

	All patients (*N*=300) *N* (% or range)
Age, years	72 (18–96)
Sex, male	193 (64)
Department	
Medical	127 (42)
Surgical	126 (42)
Infectious diseases	44 (15)
ICU	3 (1)
CCI, score	2 (0–12)
Emergency admission	241 (80)

ICU, intensive care unit; CCI, Charlson Comorbidity Index.

**Table Ⅱ:** Comparison of all patients with colonized and non-colonized peripheral venous catheters (PVCs).

Potential risk factor	Colonization, any *N*=33	No colonization *N*=267	OR	95% CI	*P*-value
Age, years					
18–69 (ref)	12	109	1	0.6–2.6	0.61
≥70	21	158	1.2		
Sex, male (%)	73	63	0.9	0.4–2.1	0.87
Department					
Medical (ref)	18	108	1		
Surgical	14	112	0.8	0.4–1.6	0.47
Infectious diseases	1	43	0.1	0.1–1	0.06
ICU		3			
CCI, score					
0–4 (ref)	27	217	1		
5–12	6	50	0.6	0.2–1.9	0.40
Admission form					
Emergency (ref)	29	211	1		
Scheduled	4	56	0.6	0.2–1.5	0.23
Dwell time at sample collection					
2 days (ref)			1		
≥3 days			0.8	0.3–2.1	0.70
Insertion site					
Cubital fossa (ref)	4	81	1		
Forearm	12	77	3.2	0.9–10.2	0.06
Hand	8	40	4.1	1.2–14.3	0.03
Foot		2			
Unknown	9	67	2.7	0.8–9.2	0.11
PVC size					
20 G (ref)	17	99	1		
22 G	3	43	0.4	0.1–1.5	0.17
18 G	3	34	0.51	0.1–1.9	0.31
17 G		1			
Size not reported	10	90	0.64	0.3–1.5	0.29
Immunocompromised					
No (ref)	29	231	1		
Yes	4	36	0.9	0.3–2.8	0.87

OR, odds ratio; CI, confidence interval; ref, reference; G, gauge; ICU, intensive care unit; CCI, Charlson Comorbidity Index.

The positive culture results were as follows: port alone, 26 (8.7%); internal lumen alone, 5 (1.7 %); and port and internal lumen, 2 (0.7%). The colonization rate was significantly higher in the injection port than in the internal lumen (*P*<0.0001). Different species of coagulase-negative staphylococci (CoNS) were found in 30 of 33 (91%) positive cultures. In two cases, indistinguishable strains were found in the port and the internal lumen (*E. faecium and S. aureus)*. The results of the PVC cultures are shown in Table III. None of the patients with a colonized PVC had a positive blood culture within ±72 h of PVC sample collection. Two patients were treated with antibiotics because of suspected PVCR infection. Both were in the non-colonized group. No patients in this study died from a PVCR infection.

**Table Ⅲ:** Characteristics of patients with colonized peripheral venous catheters (PVCs).

Patient no.	Age, years	Sex	Length of stay	CCI score	Diagnosis	Emergency admission	PVC size	PVC insertion site	Port/infusion/both	Dwell time at sample collection (days)	Micro-organisms	Number of positive cultures
14	28	Female	7	1	Budd Chiari syndrome, postoperative care	Yes	-	-	Internal lumen	>2^a^	*S. epidermidis*	1
20	35	Female	7	0	Postoperative infection after cholecystectomy	Yes	20 G	-	Internal lumen	>2^a^	*S. epidermidis, S. hominis*	2
31	43	Male	3	2	Non-ST-elevation myocardial infarction	Yes	-	Forearm	Port	3	*Rothia* spp.*, S. epidermidis*	2
40	50	Female	6	0	Paroxysmal ventricular tachycardia	Yes	20 G	Forearm	Port	2	*S. capitis, S. epidermidis, S. hominis, S. warneri*	4
52	55	Male	28	3	Liver cirrhosis caused by alcohol	Yes	20 G	-	Port	4	*S. epidermidis*	1
57	56	Male	3	0	Pulmonary embolism	Yes	20 G	Forearm	Port	3	*S. capitis*	1
59	57	Male	20	0	Ulcerative colitis	Yes	18 G	Hand	Internal lumen	7	*S. lugdunensis*	1
88	65	Male	12	6	Malignant tumour in rectum	No	-	-	Port	4	*S. epidermidis*	1
102	68	Male	16	1	Chronic leg ulceration	Yes	20 G	Cubital fossa	Port	3	*S. epidermidis*	1
104	68	Male	13	1	Atrial fibrillation	Yes	18 G	Cubital fossa	Port	2	*S. capitis*	1
105	68	Male	2	3	Atrial flutter	Yes	20 G	Forearm	Port	2	*S. hominis*	1
117	69	Male	47	6	Atherosclerotic heart disease	No	20 G	Hand	Port	2	*S. epidermidis*	1
126	70	Male	22	1	Non-ST-elevation myocardial infarction	Yes	20 G	Forearm	Port	3	*S. epidermidis*	1
130	70	Male	9	2	Heart failure	Yes	20 G	Forearm	Port	3	*S. epidermidis*	1
136	71	Female	11	3	Pulmonary hypertension	Yes	22 G	Hand	Both	4	*S. aureus, S. epidermidis*	3
147	72	Male	13	6	Malignant tumour in colon	Yes	20 G	Hand	Port	4	*S. capitis*	1
160	74	Male	3	2	Atrial fibrillation	Yes	20 G	Forearm	Port	2	*S. epidermidis, S. hominis*	2
178	76	Male	11	2	Acute appendicitis	Yes	20 G	-	Port	4	*S. hominis*	1
194	77	Female	4	1	Non-ST-elevation myocardial infarction	Yes	20 G	Forearm	Port	2	*S. hominis*	1
196	77	Male	8	2	Malignant tumour in rectum	No	20 G	Hand	Port	3	*S. epidermidis*	1
205	78	Female	11	2	Malignant tumour in rectum	No	-	-	Port	>2^a^	*S. hominis*	1
215	80	Male	8	1	Right ventricular failure	Yes	20 G	Forearm	Port	5	*S. capitis*	1
217	80	Female	7	5	Mitral insufficiency	Yes	20 G	Cubital fossa	Port	2	*S. aureus*	1
218	80	Female	5	2	Paroxysmal atrial fibrillation	Yes	-	Cubital fossa	Port	3	*S. capitis*	1
229	81	Male	6	3	Aortic valve stenosis	Yes	18 G	Hand	Port	2	*S. capitis*	1
230	81	Male	3	1	Chronic ischaemic heart disease	Yes	-	Forearm	Port	2	*S. epidermidis*	1
240	82	Male	7	4	Bradycardia	Yes	20 G	Forearm	Port	4	*S. hominis*	1
257	84	Male	6	1	Atrial flutter	Yes	-	-	Internal lumen	2	*S. capitis*	1
259	84	Male	6	3	Gastrointestinal bleeding	Yes	-	-	Port	>2^a^	*S. capitis, S. epidermidis, S. haemolyticus*	3
265	86	Male	20	8	Malignant tumour in duodenum	Yes	22 G	Hand	Both	2	*Enterococcus faecium*	2
277	88	Male	23	2	Obstipation	Yes	-	Forearm	Port	5	*S. epidermidis,* viridans streptococci	2
285	89	Female	6	2	Diverticulum in colon without perforation	Yes	-	-	Internal lumen	>2^a^	*S. hominis*	1
294	91	Male	18	6	Malignant tumour in colon transversum	Yes	22 G	Hand	Port	4	*Actinomyces radicidentis,* viridans streptococci	2

CCI, Charlson Comorbidity Index; G, gauge; -, missing data.

a The peripheral venous catheter was in place for >2 days, but the exact insertion time was not registered.

## Discussion

This study showed that 11% of PVCs were colonized with micro-organisms and that the rate of microbiological colonization was significantly higher in the injection port than in the internal lumen of the catheter. The only significant risk factor was having a ported PVC in the hand, which differs from previous findings [12]. Almost all micro-organisms found in this study were potential human pathogens.

To the best of the authors' knowledge, this is the first study to prospectively investigate the microbiological colonization of ported PVCs *in vivo*, and compare colonization of the injection port with colonization of the internal lumen. This is of importance because ported PVCs are commonly used in Scandinavia. To the authors' knowledge, only one prospective randomized study has compared complications between two different types of PVCs (open and closed) [7]. This showed that a closed system has lower risk for PVCR-BSI; however, the differences in catheter design between the ported PVC and those in this study make it difficult to compare the results.

Using NCs for PVCs without a port is recommended and commonplace in clinical practice [13]. As opposed to using an injection port, use of an NC enables disinfection of the surface prior to injection. Several studies have shown that although the colonization rate of NCs is between 20% and 50%, appropriate NC disinfection can reduce the rate substantially (0–2%) [12,14,15]. However, the difficulties in following proper disinfection routines in daily clinical practice can lead to high colonization rates and an unintended increase in PVCR-BSI [12]. Moreover, inappropriate NC design and/or low adherence to disinfection routines increases the number of catheter-related BSIs [9]. This should be compared with the port on ported PVCs, which cannot be disinfected properly prior to use. Hence, it is difficult to judge whether the present finding of a colonization rate of 8.7% in the port is higher or lower than the actual rate of colonization of NCs in clinical use. Furthermore, NCs and ported PVCs can be colonized with a biofilm on the internal lumen, and these micro-organisms are not susceptible to external disinfection [12,16].

Of the 300 sampled PVCs, seven positive cultures were found in the internal lumen, of which two had an indistinguishable bacterial strain concurrently in the port. It is possible for micro-organisms to migrate from the port to the internal lumen [16,17]. It is impossible to determine if this was the case in these two patients. In five patients, colonization of the internal lumen of the catheter was present without colonization of the port, indicating that the interior surface can be colonized by several mechanisms. The importance of these different routes must be evaluated further.

Most cultures from patients with a colonized PVC showed growth of CoNS (30/33; 90.1%), and nearly all strains could be responsible for PVCR-BSIs. The former is in accordance with previous findings [8]. None of the CoNS strains identified were found in both the port and the internal lumen. The two cases of indistinguishable strains in both locations were caused by *S. aureus* and *E. faecium*; these bacteria are known to cause more severe infections than CoNS. It is unknown whether the ability to migrate through the port to the internal lumen differs between different micro-organisms, and this warrants further research.

It has also been shown that introducing bundles with high adherence can decrease the frequency of PVCR-BSI, and even decrease infection-related mortality [4,6]. It is, therefore, of great importance to determine which factors in a bundle are important to decrease PVCR-BSIs successfully. In the authors' opinion, the question regarding the best PVC design in terms of infection prevention remains unanswered. Additionally, newer PVCs are often more expensive than their predecessors, and introducing PVCs without any evidence from randomized controlled trials regarding their benefit may lead to unmotivated costs. Given the different challenges with disinfection of ports and NCs, it is unclear from the findings which device should be preferred.

Interestingly, patients in the infectious diseases department had an odds ratio for colonization of 0.1 (95% confidence interval 0.1–1; *P*<0.06) compared with those in the medical department. This may be due to differences in adherence to hygiene routines or higher use of antibiotics in the infectious diseases department.

These data suggest that ported PVCs with associated cleaning difficulties may have higher colonization rates than NCs in which appropriate disinfection adherence is upheld (8.7% vs 0–2%). However, this must be related to the hospital setting as low adherence to disinfection routines may lead to high NC colonization rates (up to 20–50%). In view of the study findings, it may be beneficial to avoid placing PVCs in patients' hands to further limit the risk of colonization. The 72-h replacement routine should also be challenged, as the study data did not show a significantly higher colonization rate for the 50% of PVCs that were removed >72 h after insertion. The latter suggestion would potentially decrease patient discomfort due to lower frequency of PVC insertions. Together, these changes could enable continuation of the use of ported PVCs. Overall, it is believed that adequate hygiene routines and firm adherence to them are the most important factors for sustaining low rates of colonization and PVCR-BSIs.

This study has some limitations. First, as PVCs were sampled *in situ*, it was not possible to perform tip cultures that could have provided more information about the migration of micro-organisms along the external part of the PVC. Second, the clinical impact of port colonization in relation to PVCR-BSI is unknown. Third, in this study, the institutional routine was to replace PVCs every 72 h. This may have introduced bias into the colonization data in favour of lower colonization rates. However, half of the PVCs in this study had a longer dwell time than 72 h, and reflect the colonization rate of dwell times between 4 and 8 days. Fourth, this study emphasized the question of whether the port is a problem. The observational study design may have modified clinical practice during the study, leading to less use of the port, which may have led to less flushing, possibly leaving more bacteria in the port ready to be caught on the swab compared with the internal lumen. Fifth, for practical reasons, the cultures were not performed on PVCs in consecutive patients, introducing a risk of selection bias. Finally, the departments included in this study have a vested interest in knowledge and research about PVC hygiene routines. Therefore, the results may represent a lower rate of colonization compared with that observed in other general departments of the hospital. In the authors' opinion, there is an urgent need for a randomized controlled trial comparing ported PVCs and PVCs with NCs investigating PVCR-BSI as the primary endpoint.

In conclusion, this study showed colonization of micro-organisms in 11% of ported PVCs, and the vast majority (8.7%) were found in the injection port. This should be considered and related to other types of PVCs when choosing a ported PVC for insertion and use.

## Author contributions

Study concept: all authors.

Preparation of the protocol: all authors.

Principal investigator: DJ.

Drafting of the manuscript: DJ.

Application for ethical approval and funding: DJ.

Statistical analyses with support of independent statisticians: KT and DJ.

Responsible for recruitment of patients: DJ.

Data collection: DJ and SM.

All authors helped prepare the final manuscript and agreed to be accountable for all aspects of the work, thereby ensuring that questions related to the accuracy or integrity of any part of the work were appropriately investigated and resolved.

## Conflict of interest statement

None declared.

## Funding source

The study was supported by 10.13039/501100009752Futurum, The Academy for Healthcare, Jönköping County Council, Sweden (Grant No. 519811).
